# Supplementary material for: Complexome profiling of the Chlamydomonas psb28 mutant reveals TEF5 as an early PSII assembly factor
Source: Plant Cell. 2025 Mar 17;37(6):koaf055. doi: 10.1093/plcell/koaf055 (PMC12164590; doi:10.1093/plcell/koaf055)
Supplement: koaf055_Supplementary_Data [file koaf055_supplementary_data.zip › psb28_interactive.zip!/plots/intensity/qualityPlots/peptides/PRPS20_Cre12.g494750.t1.2.html]

### Cre12.g494750.t1.2

Trivial name: PRPS20  
SeqCoverage [median]: 38.00  
Mass: 18.25 kDa  
  
 

| Sequence | MissedCleavage | Mass [Da] | wtIntensity | muIntensity | Score |
| --- | --- | --- | --- | --- | --- |
| ALLSEER | 0 | 816.43 | 0.00 | 354.88 | 76.03 |
| LAETLVAAPSKTEEEVKNLEK | 2 | 2298.23 | 0.00 | 1092.28 | 34.01 |
| LISEAYTEIDK | 0 | 1280.65 | 0.00 | 352.18 | 54.61 |
| LISEAYTEIDKAVVK | 1 | 1677.92 | 0.00 | 878.47 | 55.72 |
| TVLMAAGLYKPAADSPDFAR | 1 | 2093.06 | 0.00 | 1144.42 | 48.48 |
|  |  |  | 0.0 | 3822.2 |  |
